# The Predictive Value of Dyadic Coping in the Explanation of PTSD Symptoms and Subjective Well-Being of Work Accident Victims

**DOI:** 10.3389/fpsyg.2018.01664

**Published:** 2018-09-07

**Authors:** Susana Lameiras, Alexandra Marques-Pinto, Rita Francisco, Susana Costa-Ramalho, Maria Teresa Ribeiro

**Affiliations:** ^1^Faculdade de Psicologia, Centro de Investigação em Ciências Psicológicas, Universidade de Lisboa, Lisbon, Portugal; ^2^Faculdade de Psicologia e de Ciências da Educação, Universidade de Coimbra, Coimbra, Portugal; ^3^Faculdade de Ciências Humanas, Universidade Católica Portuguesa, Lisbon, Portugal; ^4^Católica Research Centre for Psychological, Family and Social Wellbeing (CRC-W), Universidade Católica Portuguesa, Lisbon, Portugal

**Keywords:** work accidents, dyadic coping, PTSS, subjective well-being, couples

## Abstract

**Objective:** Work accidents may be considered dyadic stressors in so far as they not only affect the worker, but also the couple’s relationship. Dyadic coping, as the process by which couples manage the stress experienced by each partner, can strengthen individual health and well-being as well as couple relationship functioning. Accidents at work have progressively been studied from a perspective that focuses on their negative effects on PTSS, anxiety, and depression. However, to a large extent, the dyadic coping processes and results following a work accident are still to be identified and clarified. In this study, we examined the predictive value of dyadic coping in the explanation of PTSS and subjective well-being of work accident victims.

**Method**: This study comprised a sample of 62 individuals involved in work accidents within the last 24 months (61.3% males) and their partners (*N* = 124; *M* = 46.25 years, *SD* = 11.18). All participants responded to the Dyadic Coping Inventory and the work accident victims also answered the PTSD Checklist – Civilian (PCL-C) and the Mental Health Continuum – Short Form (MHC-SF). Two hierarchical multiple regression analyses were performed using two different variable set models: Model 1 comprised the control variables gender and age, and Model 2 included the workers’ and the partners’ dyadic coping variables.

**Results**: Results showed that dyadic coping reported by both workers and their respective partners (Model 2) was a significant predictor of workers’ PTSS (*p* < 0.01) and subjective well-being (*p* < 0.001), explaining 31.2% of the variance in PCL-C and 68.7% in MHC-SF results. More specifically, the partners’ supportive dyadic coping (by the self) and delegated dyadic coping (by the partner) were significant predictors of the workers’ lower PTSS and virtually all the dyadic copying strategies of both the workers’ and their partners’ were significant predictors of the workers’ higher subjective well-being.

**Conclusion**: Dyadic coping of both the workers and their partners predicts the workers’ PTSS and subjective well-being. These findings point to the need to work with couples who have experienced a work accident, with a view to improving the workers’ mental health outcomes.

## Introduction

Work accidents experienced by a member of a couple may be thought of as dyadic stressors since they also affect the partner and the couple relationship functioning. The present research focused on the dyadic coping processes that sustain adaptive efforts following a work accident, with a view to understanding the predictive value of dyadic coping in the explanation of work accident victims’ post-traumatic stress disorder symptoms (PTSSs) and subjective well-being.

A work accident is defined in the European Statistics on Accident at Work as “a discrete occurrence in the course of work which leads to physical or mental harm” (Eurostat, 2013, p. 5). A work accident is considered to occur in the course of work when it takes place during the time spent at work or while performing an occupational activity. Accidents at work occur quite frequently. It is estimated that 3,211,706 accidents at work occurred in the European Union in 2015, causing at least 1 day of absenteeism (Eurostat, 2018), and that in the same year 208,457 work accidents occurred in Portugal (Pordata, 2018).

Empirical evidence suggests that, in specific circumstances, accidents at work may be traumatic events and possibly lead to the development of PTSD symptoms, involving an involuntary re-experience of the accident and avoidance of related memories (Gustafsson and Ahlström, 2004). The development of PTSD symptoms may emerge in the aftermath of work accidents involving injuries such as burns or amputation, and also following accidents at work in which there is no actual physical harm such as an armed robbery, attack, or hijack (MacDonald et al., 2003; Schaefer et al., 2012).

When work accidents happen to a married or cohabiting individual, it can be a stressful and challenging experience not only for the worker but also for his/her partner and the relationship itself. According to the systemic-transactional model of Bodenmann (1995), stressors are dyadic when they are directly related to the couple, namely when both members are dealing with the same stressor, and indirectly related when the stress of one of the partners affects both of them (Kramer et al., 2005).

In the same vein, married (or cohabiting) victims of work accidents do not cope with the situation alone, and the relationship with their partner may be their primary coping resource. Dyadic coping is a construct, based on Lazarus and Folkman’s (1984) Transactional Stress Theory, which defines the efforts on the part of one or both partners to deal with stress and to create or restore the psychological, physical, and social homeostasis of the couple relationship (Bergstraesser et al., 2015). According to this perspective, dyadic coping may be regarded as a form of interaction between partners when they are dealing with a stressful situation, and both individuals and their respective partners are independently and mutually influenced by each other in their adjustment processes (Bodenmann, 1995). The two main goals of dyadic coping are to reduce the stress for each member of the couple, and to maintain the quality of the relationship. Research over recent decades has confirmed the significant value of dyadic coping in predicting couples’ relationship satisfaction and subjective well-being (e.g., Bodenmann et al., 2011).

The dyadic coping process is activated when one of the partners communicates the stressor to the other, whether verbally or non-verbally. The other member of the couple receives and interprets the signs of stress and responds with some type of dyadic coping (Hansen et al., 2015). Bodenmann proposes three types of dyadic coping: supportive dyadic coping, which is related to the stress response behaviors of one partner toward the other, such as assistance, encouragement and emotional support; delegated dyadic coping in which one partner is primarily affected by the stressful event and asks the other to take over several tasks to reduce his or her levels of stress; and common dyadic coping, which is defined as couple implemented behaviors to actively collaborate in problem solving, joint decision making and seeking ways of reducing the stress together (e.g., Costa-Ramalho et al., 2017). Some types of dyadic coping, like hostile, ambivalent and superficial, can also be negative (Bodenmann et al., 2006). Studies and reviews regarding the impacts of different types of dyadic coping are presented in the following paragraphs.

Traditional approaches toward coping and adjustment to adversity have tended to focus on the individual, however in recent decades an increasing number of studies have pointed to the profound impacts of traumatic events on both members of the couple and their relationship, and have sought to examine partners’ dyadic coping efforts. In the case of chronic illness, for instance, research has shown that couples are challenged to re-establish communication patterns, roles and responsibilities (Bodenmann et al., 2004; Gabriel et al., 2016). Within the scope of serious illness, more positive dyadic coping styles correlate with higher relationship satisfaction, higher relationship quality, and higher quality of life (Bodenmann, 2000; Revenson et al., 2005). Several other reviews have highlighted the importance of couples’ intervention in order to boost dyadic coping strategies while dealing with cancer, and have revealed the positive repercussions of this approach in terms of psychosocial adjustment and relationship functioning not only in patients but also in their partners (Badr and Krebs, 2013). Traa et al. (2015) also showed the importance of adequate dyadic coping efforts, such as stress communication, supportive behaviors, and positive dyadic coping, for the maintenance and improvement of relationship functioning in couples coping with cancer. Other reviews have shown that traumatic events, such as the loss of a child (Albuquerque et al., 2015), terror attacks (Gilber et al., 2011) and natural catastrophes like earthquakes (Marshall et al., 2017), can bring some couples closer while others demonstrate significant adjustment problems that are linked to conflict and may result in divorce (e.g., Badr and Acitelli, 2017). Adaptive processes mediate the effects of personal characteristics and stressful events on marital adjustment. Some behaviors promote marital resilience, such as communication, cooperation, and mutuality and the absence of these factors may undermine the couple’s adjustment when facing a stressful event (Karney and Crown, 2007).

Moreover, Bodenmann (2005) suggested that different types of dyadic coping strategies may have positive or negative effects on marital functioning. In the case of the former, these strategies may include problem-focused or emotion-focused supportive dyadic coping, common dyadic coping and delegated dyadic coping, while negative effects may result from hostile, ambivalent, and superficial dyadic coping. Nevertheless, the effectiveness of different forms of coping may be hard to establish *a priori*, since it depends on a host of variables such as the nature of the stressors, the pattern of coping strategies used to confront them or the outcome variables selected to evaluate their effectiveness (Folkman and Moskowitz, 2004). For instance, as regards mental health outcomes, Lafaye et al. (2014) showed that when patients or spouses used emotion-focused coping strategies they experienced higher levels of anxiety and depressive symptoms. Likewise, Manne and Badr (2008) found that in couples in which one partner is chronically ill, some forms of positively intended dyadic coping such as excessive kindness, concern, and support may be dysfunctional as they paralyze the patient’s own coping efforts.

Accidents at work have progressively been studied from a trauma perspective, focusing on the negative psychopathological consequences for the worker, with particular emphasis on PTSD, anxiety, and depression (MacDonald et al., 2003). However, dyadic coping processes, which sustain adaptive effort in the aftermath of an accident, and their relationship with the mental health and subjective well-being of work accident victims, are yet to be investigated.

Gender and age often function as demographic variables in trauma research, and, to date, the results of a number of epidemiological studies have pinpointed both gender and age differences in PTSD, noting different developmental distributions of PTSD for men and women across their life courses. In a more detailed way, some studies point to the fact that women present a higher level of PTSD throughout life depending on the exposure to stress factors (i.e., poverty, lack of social support, partner violence). Overall, research has demonstrated that PTSD is more dependent on social, economic, cultural, and historical factors than dependent on age (Ditlevsen and Elklit, 2010).

Therefore, the aim of this study is to understand the predictive value of dyadic coping, reported by workers who have suffered a work accident and their partners in the explanation of the worker’s PTSS and subjective well-being, while controlling for the effects of the worker’s gender and age. Inasmuch no empirical studies were found on dyadic coping processes following a work accident and their relationships with mental health and well-being and it is hard to establish *a priori* the effectiveness of different forms of dyadic coping, the present study set out to analyze these relationships in an exploratory manner.

This study may contribute to further the knowledge on workers who have suffered a work accident and their partners, a population the scientific literature has largely forgotten. More specifically, it may contribute to detail the consequences of work accidents with regard to post-traumatic stress symptoms and subjective well-being, as well as the role played by the couple dyadic coping efforts in the prediction of those consequences. Moreover this study may provide empirical evidence with a view to stimulating interventions geared toward getting the best out of the workers and their partners’ dyadic coping when facing a work accident.

## Materials and Methods

### Participants

The sample comprised 62 individuals involved in a work accident within the last 24 months (61.3% males) and their partners (*N* = 124) aged between 19 and 68 years (*M* = 46.25; *SD* = 11.18). Participants had been married or cohabiting from 1 to 41 years (*M* = 16.60; *SD* = 11.50). The majority of participants had children (82.3%) and lived in Portugal, mostly in the central region (60.5%). In terms of schooling, 42.8% had not completed secondary education, 26.6% had completed secondary education, and 25.8% held a university degree. The majority of the respondents were Portuguese (96%).

### Procedure

Data were collected between January 2016 and December 2017 in the medical clinics of Insurance Companies in Lisbon and Oporto 1 month or more after the work accident had occurred. Paper and pencil questionnaires were given by nurses and physiotherapists to individuals who had had a work accident and who were in a marital relationship (marriage or cohabitation) and, at the same point in time, to their partners. The workers’ participation rate is unknown but the partners’ participation rate was of 100%. Workers answered the Dyadic Coping Inventory (DCI), the Mental Health Continuum – Short Form (MHC-SF), and the PTSD Checklist – Civilian (PCL-C) and their partners answered the DCI. The questionnaires of both workers and their partners received a similar code number to enable their pairing while preserving the anonymity of the answers. The participants were volunteers and no financial remuneration was provided for their participation. The time taken to complete the questionnaires varied between 30 min for the workers and 10 min for their partners. Anonymity and confidentiality were guaranteed to the participants, as was the data being used strictly for research purposes. Both the workers and their partners were informed of the main objectives of the research and signed an informed consent form. The study was approved by the Ethics Committee of the University where the study is in course.

### Measures

#### Dyadic Coping Inventory

The Portuguese adaptation by Vedes et al. (2013) of the DCI by Bodenmann (2008) was used. The DCI is a self-report inventory of dyadic coping with 37 items (e.g., *My partner shows empathy and understanding to me*), scored using a five point Likert scale ranging from 1 (*very rarely*) to 5 (v*ery often*). The original DCI has nine subscales: Stress Communication by Self, Stress Communication by Partner (or other), Supportive DC by Self, Supportive DC by Partner, Negative DC by Self, Negative DC by Partner, Delegated DC by Self, Delegated DC by Partner and Joint DC in addition to a global score. The Joint DC subscale describes the coping behavior shown by both partners, while the first eight subscales refer to the coping behavior of the self and partner separately. In the present study, all sub-scales presented acceptable to good alphas in both the workers’ sample and the partners’ sample, except in the sub-scales Stress Communication by Self and Negative DC by Self in the workers’ sample, and Delegated DC by Self in the partners’ sample which presented low alphas and were therefore excluded from the analysis.

#### PTSD Checklist – Civilian

The PCL-C (Weathers et al., 1993; Portuguese version by Marcelino and Gonçalves, 2012) is a standardized self-report scale for PTSD symptoms comprising 17 items (e.g., *Repeated, disturbing memories, thoughts, or images of a stressful experience from the past?*) to be answered on a five point Likert scale from 1 (*not at all*) to 5 (*extremely*). In the current study, the PCL-C instructions asked the participants to give their answers bearing in mind specifically the actual work accident they had suffered. The PCL-C is usually used as a continuous measure of PTSD symptom severity, whereby a global score may be obtained simply by adding up the scores for all 17 items, with values ranging from 17 to 85, and higher scores indicating more severe PTSD symptoms. Although the PCL-C Portuguese version is still not in conformity with DSM-V, which has add a new dimension to the criteria for diagnosis, in this study only the global score was used (American Psychiatric Association, 2000). The PCL-C has demonstrated strong psychometric properties namely in the Portuguese validation by Marcelino and Gonçalves (2012; e.g., alpha value of 0.94), and an excellent alpha value of 0.95 was obtained in the workers’ sample in this study.

#### Mental Health Continuum – Short Form

The MHC-SF (Keyes, 1998; Portuguese version by Carvalho et al., 2016), consists of 14 items (e.g., *During the past month how often have you felt happy?*) ranging from 1 (*never*) to 6 (*every day*), reflecting emotional, psychological and social well-being. The emotional well-being subscale is defined in terms of positive affect or satisfaction with life, psychological well-being refers to self-acceptance, environmental mastery, positive relations with others, personal growth, autonomy and purpose in life and social well-being is related to social contribution, social integration, social actualization, social acceptance, and social coherence. Three specific scores may be estimated, one for each well-being dimension, as well as a global score (ranging from 14 to 84), reflecting subjective well-being as a whole. The Portuguese version of the MHC-SF used in the present study demonstrated strong psychometric properties (e.g., alpha value of 0.90 for the total questionnaire; Carvalho et al., 2016). In this study, the global score was used and an excellent alpha value of 0.90 was attained.

### Data Analysis

The data were analyzed using SPSS, version 25. Descriptive statistics (means and standard deviations) and Pearson correlations were computed for the study variables. Regression analyses were used to assess the predictive value of dyadic coping in the explanation of workers’ PTSS and subjective well-being, while controlling for the effects of their gender and age. More specifically, two hierarchical multiple regression analyses (enter method) were performed using two different variable set models: to control for the effects of demographic variables, the gender and age of the workers were introduced in Model 1. Model 2 included the workers’ and partners’ dyadic coping variables. Prior to conducting the hierarchical multiple regressions, the relevant assumptions of this statistical analysis were verified through the graphical analysis of the studentized residuals, the Durbin–Watson statistic and the VIF statistic. The Durbin–Watson test statistic value found (*d* = 1.77) falls inside the range of 1.5 to 2.5, therefore indicating that the data are not autocorrelated (Field, 2009). One predictor (i.e., workers’ Supportive DC by Partner) with VIF (= 22.62) above the acceptable limits (Field, 2009) was removed from the model to avoid the effects of multicollinearity. Those with *p* < 0.05 were considered to be significant effects.

## Results

**Table 1** presents the means, standard deviations, inter- correlations, and internal consistencies (Cronbach’s alpha) for the measures used in this study (PCL-C, MHC-SF, and DCI for the workers’ sample and DCI for the partners’ sample).

**Table 1:** Descriptive statistics, internal consistency (**in bold**) and inter correlations for study variables.

Measure	*M*	*SD*	1	2	3	4	5	6	7	8	9	10
(1) **PTSD Checklist – Civilian**	45.61	18.64	**0.95**									
(2) **Mental Health Continuum**	53.67	15.65	–0.554^∗∗^	**0.90**								
**Dyadic Coping Inventory** (worker)												
(3) Stress Communication (p)	14.54	3.41	–0.337^∗∗^	0.381^∗∗^	**0.76**							
(4) Supportive DC (s)	18.77	3.54	–0.267^∗^	0.441^∗∗^	0.479^∗∗^	**0.79**						
(5) Supportive DC (p)	18.70	3.93	–0.272^∗^	0.467^∗∗^	0.668^∗∗^	0.691^∗∗^	**0.79**					
(6) Delegated DC (s)	7.00	2.06	–0.426^∗∗^	0.562^∗∗^	0.274^∗^	0.448^∗∗^	0.307^∗^	**0.78**				
(7) Delegated DC (p)	6.61	2.24	0.001	0.299^∗^	0.388^∗∗^	0.649^∗∗^	0.786^∗∗^	0.156	**0.69**			
(8) Negative DC (p)	9.38	3.38	0.043	–0.226	–0.300^∗^	–0.493^∗∗^	–0.371^∗∗^	–0.061	–0.455^∗∗^	**0.74**		
(9) Joint DC	16.48	3.62	–0.132	0.576^∗∗^	0.394^∗∗^	0.732^∗∗^	0.609^∗∗^	0.442^∗∗^	0.587^∗∗^	–0.456^∗∗^	**0.83**	
**Dyadic Coping Inventory** (partner)												
(3) Stress Communication (s)	14.77	2.81	–0.322^∗∗^	0.464^∗∗^	**0.67**							
(4) Stress Communication (p)	14.09	3.13	–0.189	0.322^∗^	0.170	**0.63**						
(5) Supportive DC (s)	19.22	3.02	–0.163	0.028	0.348^∗^	0.489^∗∗^	**0.73**					
(6) Supportive DC (p)	17.64	4.90	–0.120	0.382^∗∗^	0.360^∗∗^	0.658^∗∗^	0.386^∗∗^	**0.88**				
(7) Delegated DC (p)	6.58	1.94	–0.310^∗^	0.587^∗∗^	0.384^∗∗^	0.452^∗∗^	0.022	0.613	**0.77**			
(8) Negative DC (s)	8.29	3.64	–0.003	–0.055	–0.186	0.012	–0.241	–0.316^∗^	0.017	**0.78**		
(9) Negative DC (p)	9.80	3.41	0.127	–0.209	0.146	–0.497^∗∗^	–0.186	–0.430^∗∗^	–0.235	0.224	**0.70**	
(10) Joint DC	17.06	3.23	0.509	0.443^∗∗^	0.352^∗∗^	0.535^∗∗^	0.370^∗∗^	0.629^∗∗^	0.629^∗∗^	0.048	–0.150	**0.79**

*DC, Dyadic Coping; *M*, mean; *SD*, standard deviation; ^∗^*p* < 0.05, ^∗∗^*p* < 0.01, ^∗∗∗^*p* < 0.001.*

In the sample of work accident victims, the global scores for PTSS and for subjective well-being attained mean values above the respective midpoint of each of the distribution scores (minimum of 17 and maximum of 85; minimum of 14 and maximum of 84, respectively).^1^

The workers’ PTSS presented a significant strong negative correlation with the workers’ subjective well-being, significant weak to moderate negative correlations with the workers’ Stress Communication (partner), Supportive DC (both self and partner) and Delegated DC (self), and also significant moderate to strong negative correlations with the partners’ Stress Communication (self) and Delegated DC (partner). On the other hand, the workers’ subjective well-being presented significant moderate to strong positive correlations with all the workers’ DCI subscales, except Negative DC (partner), and also with the partners’ Stress Communication (both self and partner), Supportive DC (partner), Delegated DC (partner), and Joint DC.

In the workers’ sample, the DCI subscales presented significant moderate to strong positive inter-correlations, with the exception of the correlations between Delegated DC (self) and Delegated DC (partner) and Negative DC (partner), which were weak and non-significant, and the correlations between Negative DC (partner) and the other subscales which were also negative. In the partners’ sample Stress Communication (both self and partner) presented significant moderate to strong positive correlations with Supportive DC (both self and partner), Delegated DC (partner), and Joint DC. This last subscale also revealed significant moderate to strong positive correlations with Supportive DC (both self and partner) and Delegated DC (partner). Supportive DC (self) presented significant moderate positive correlations with Supportive DC (partner). Negative DC (partner) presented significant moderate to strong but negative correlations with Stress Communication (partner) and Supportive DC (partner); similarly, Negative DC (self) showed a significant moderate negative correlation with Supportive DC (partner).

Two two-step hierarchical multiple regression analyses were conducted with PTSS and subjective well-being as the dependent variables. The gender and age of the victims were entered at step one of the regression, to control for demographic effects (Model 1), and the workers’ and partners’ dyadic coping were entered at step two (Model 2). The regression statistics are presented in **Table 2**.

**Table 2:** Results of the hierarchical multiple regression analyses in the predictors of the workers’ PTSD and subjective well-being.

	PTSD						Well-Being					
	Model 1			Model 2			Model 1			Model 2		
	*B*	*SE B*	*β*	*B*	*SE B*	*β*	*B*	*SE B*	*β*	*B*	*SE B*	*B*
Workers’ Gender	3.962	4.908	0.104	–5.837	5.942	–0.154	–4.399	3.976	–0.138	1.752	3.364	0.055
Workers’ Age	–0.100	0.219	–0.059	–0.182	0.263	–0.107	0.353	0.177	0.249*	0.588	0.149	0.414***
Workers’ Stress Communication by P.				1.128	1.362	0.207				–0.044	0.771	–0.010
Workers’ Supportive DC by S.				–2.631	1.445	–0.500				–0.006	0.818	–0.001
Workers’ Delegated DC by S.				–2.197	1.712	–0.243				0.623	0.969	0.082
Workers’ Delegated DC by P.				2.697	1.457	0.324				1.813	0.825	0.260*
Workers’ Negative DC by P.				–0.247	0.981	–0.045				0.641	0.555	0.139
Workers’ Joint DC				–0.427	1.276	–0.083				1.692	0.723	0.392*
Partners’ Stress Communication by S.				–1.607	1.361	–0.243				1.182	0.771	0.212
Partners’ Stress Communication by P.				–1.207	1.517	–0.203				0.706	0.859	0.142
Partners’ Supportive DC by S.				–3.409	1.414	–0.553*				–1.591	0.800	–0.308*
Partners’ Supportive DC by P.				3.149	1.120	0.828**				–0.740	0.634	–0.232
Partners’ Delegated DC by P.				–8.481	2.901	–0.885**				4.577	1.643	0.569**
Partners’ Negative DC by S.				0.712	0.809	0.139				–0.693	0.458	–0.161
Partners’ Negative DC by P.				0.051	0.874	0.009				–0.954	0.495	–0.208
Partners’ Joint DC				4.136	1.319	0.718**				–0.828	0.747	–0.171
*R^2^*		0.015			0.492			0.083			0.769	
*F* for change in *R^2^*		0.443			3.021**			2.672			9.547***
Adjusted *R^2^*		–0.019			0.312			0.052			0.687	
*F*		0.443			2.726**			2.672			9.365***	

*P., Partner; S., Self; DC, Dyadic Coping; ^∗^*p* < 0.05, ^∗∗^*p* < 0.01, ^∗∗∗^*p* < 0.001.*

The hierarchical multiple regressions results revealed that, at step one, the demographic variables (Model 1) did not contribute significantly to the regression models of either the workers’ PTSS, *F*(2,59) = 0.443, *p* > 0.05 or subjective well-being *F*(2,59) = 2.672, *p* > 0.05.

Entering the workers’ and partners’ dyadic coping variables explained 31.2% of the variance in the workers’ PTSS, and this model (Model 2) was significant, *F*(16,45) = 2,726, *p* < 0.01. When all the independent variables were included in this final step of the regression model, none of the demographic variables or the workers’ dyadic coping variables were significant predictors of the workers’ PTSS. In contrast, the partners’ dyadic coping variables, Supportive DC by Self and Delegated DC by Partner, were significant negative predictors of the workers’ PTSS, and the partners’ dyadic coping variables Supportive DC by Partner and Joint DC were significant positive predictors of the workers’ PTSS.

Finally, the introduction of the workers’ and partners’ dyadic coping variables explained 68.7% of the variance in the workers’ subjective well-being, and this model (Model 2) was significant, *F*(16,45) = 9,365, *p* < 0.001. When all the independent variables were included in Model 2, the age of the workers, the workers’ Delegated DC by Partner and Joint DC and the partners’ Delegated DC by Partner were significant positive predictors of the workers’ subjective well-being, while the partners’ Supportive DC by Self was a significant negative predictor of the workers’ subjective well-being.

## Discussion

The main aim of the present study was to understand the predictive value of dyadic coping, of both workers and their partners, following a work accident, in the explanation of the workers’ PTSS and subjective well-being.

The workers’ PTSS and subjective well-being were negatively correlated, as expected, in accordance with the current literature (Berle et al., 2018). As for the relations between dyadic coping and PTSS, results showed that the higher the use of some types of dyadic coping by the workers who had suffered a work accident, such as Stress Communication (partner), Supportive DC (both self and partner) and Delegated DC (self), and of Stress Communication (self) and Delegated DC (partner) by the partners, the lower the PTSS levels. Regarding the relations of dyadic coping with the workers’ subjective well-being, results also revealed that the higher the workers’ and their partners’ own dyadic coping strategies, with the exception of Negative DC, the higher their subjective well-being. Taken together, these results support the notion that in a dyadic stress situation, both members of the couple unleash coping efforts to maintain the functioning of the relationship (Bodenmann, 2000; Revenson et al., 2005; Badr and Krebs, 2013; Traa et al., 2015). Furthermore, they are consistent with studies of the last decade, confirming that dyadic coping is significantly related to relationship satisfaction and subjective well-being (Bodenmann et al., 2011). Positive dyadic coping is actually regarded as a central dimension to relationship quality and the partner’s well-being, as it enhances mutual trust, respect, commitment, and a sense of the relationship being comforting and supportive (Bodenmann, 2000).

Contrary to previous epidemiological studies pinpointing both gender and age differences in PTSS (Ditlevsen and Elklit, 2010), with women usually having a higher risk than men of developing PTSS after a traumatic event (Olff, 2017), in this study these demographic variables did not predict significantly the workers’ PTSS, although age proved to be a positive significant predictor of their subjective well-being. Results of prior research are inconsistent with regard to the relationships between subjective well-being and age or gender, and these relations appear to be dependent on various psychological and cultural features (Lucas and Gohm, 2000).

The dyadic coping variables, of both the workers and their partners, explained 31.2% of the workers’ PTSS, and 68.7% of their subjective well-being. These results are in line with previous literature pointing to the positive repercussions of dyadic coping strategies used by couples to deal with cancer, in terms of the patients’ psychosocial adjustment and relationship functioning (e.g., Badr and Krebs, 2013). Similarly, recent research, for instance in the context of severe illness of one partner, has highlighted that, in general, more positive dyadic coping styles correlate with higher relationship quality and satisfaction, but also with higher quality of life (Bodenmann, 2000; Revenson et al., 2005; Meier et al., 2011).

Furthermore, although the workers’ demographic and dyadic coping variables were not significant predictors of their PTSS, the partner’s Supportive DC (self) and Delegated DC (partner) were negative predictors, and Supportive DC (partner) and Joint DC were positive predictors of the workers’ PTSS. Additionally, all dyadic coping strategies were significant positive predictors of the workers’ subjective well-being, with the exception of the partners’ Supportive DC (self) that appeared to be a negative predictor. Globally, these results reveal the partners’ dyadic coping strategies as a set of efforts to deal with the work accident stressor of the workers (Bodenmann, 1995) and are consistent with a recent review on severe diseases (Traa et al., 2015), which showed the importance of stress communication, supportive behaviors, and positive dyadic coping for the maintenance or enhancement of relationship functioning in couples coping with cancer. On the other hand, although Bodenmann (2005) suggested that supportive and common dyadic coping are positive forms of coping, the present results of the predictive value of the partners’ supportive and joint dyadic coping are more in line with previous studies describing the negative effects of positively intended dyadic coping strategies (e.g., Manne and Badr, 2008). Thus, they reinforce the claim that coping functions do not reveal anything *a priori* as to what the effects of a specific type of coping will be (Folkman and Moskowitz, 2004).

## Conclusion

A work accident can be a stressor event not only for the worker but also for his/her partner, and the strategies used by both members of the couple may prove to be inefficient when they connect with higher levels of PTSS and lower levels of workers’ subjective well-being. On the other hand, couples manage to use dyadic coping strategies that allow the workers to experience lower levels of PTSS and higher subjective well-being. These results may inform future intervention efforts following a work accident and highlight the importance of couple intervention with a view to promoting the use of dyadic coping strategies with positive effects on workers’ mental health.

## Limitations

Despite the relevance of the results for theory and intervention in dyadic coping and trauma, this study presents several limitations. The first limitation regards the sample’s small size and heterogeneity in terms of length of relationship and of gender distribution. Research has shown differences related to gender (and age) in PTSS levels (Ditlevsen and Elklit, 2010; Olff, 2017), which were not found in this study, possibly due to the fact that the majority of participants were men. Thus, in future research, it is important to ensure a greater balance between the gender distribution of participants. Secondly, dyadic coping following a traumatic event has been studied using other outcome variables related to marital functioning, such as conjugal satisfaction (Bodenmann, 1995, 2000), which were not considered in the present study on the trauma of work accidents and deserve to be studied in future research. Thirdly, although a convenience sample was used, not knowing the participation rate of the workers is another limitation of the study, raising issues regarding the sample’s representativeness. Fourthly, the workers’ and partners’ perceptions of the severity of the work accident were not taken into account in the present study and future studies should control for the effect of this variable when assessing the predictive value of different types of dyadic coping strategies in the mental health of the victims of a work accident. Similarly, future research on this topic should also control for the effect of post-trauma problems with insurance companies on the mental health and subjective well-being of the victims. Additionally, as the current study is cross-sectional, reverse prediction relations between the variables can’t be ruled out and PTSS and subjective well-being may have contributed to the increased/decreased use of the different dyadic coping strategies. Finally, the one-time evaluation design used in this study also entails a high risk of not controlling for some variables, and consequently it would be of great relevance to investigate the dyadic coping efforts after a work accident using a longitudinal study design. Therefore, evaluation across time of subjective well-being, PTSS, and dyadic coping strategies should be considered in future studies, with the further aim of designing well-grounded therapeutic guidelines.

## Ethics Statement

This study was carried out in accordance with the recommendations of the Ethics Committee of the Faculty of Psychology, University of Lisbon. All participants gave written consent in accordance with the Declaration of Helsinki.

## Author Contributions

SL prepared the study design, organized the sample recruitment, collected data, and contributed to the writing of the manuscript’s introduction, methods, results, discussion, prepared the tables and references sections. AM-P prepared the study design, performed statistical analyses and prepared the tables, contributed to the writing of the manuscript’s introduction, results, and discussion. RF prepared the study design, performed statistical analyses and contribute to the writing of the introduction, methods, results, and discussion sections. SC-R contributed to write all sections of the manuscript. MR contributed to write all sections of the manuscript. All authors reviewed and approved manuscript for publication.

## Conflict of Interest Statement

The authors declare that the research was conducted in the absence of any commercial or financial relationships that could be construed as a potential conflict of interest.

## Footnotes

1 There are no studies regarding the cutoff points for the Portuguese population, but using the criteria provided by the National Center for PTSD (National Center for Posttraumatic Stress Disorder [NCPTSD], 2018) for the general population (30–35) and for a specialized medical clinic population (36–44), 36 and 28 participants in this research, respectively, would be above the cutoff point.
